# The Effects of Age, Organized Physical Activity and Sedentarism on Fitness in Older Adults: An 8-Year Longitudinal Study

**DOI:** 10.3390/ijerph17124312

**Published:** 2020-06-16

**Authors:** Alejandro Gomez-Bruton, David Navarrete-Villanueva, Jorge Pérez-Gómez, Sara Vila-Maldonado, Eva Gesteiro, Narcis Gusi, Jose Gerardo Villa-Vicente, Luis Espino, Marcela Gonzalez-Gross, Jose A. Casajus, Ignacio Ara, Alba Gomez-Cabello, German Vicente-Rodríguez

**Affiliations:** 1 GENUD (Growth, Exercise, NUtrition and Development) Research Group, University of Zaragoza, 50009 Zaragoza, Spain; dnavarrete@unizar.es (D.N.-V.); joseant@unizar.es (J.A.C.); agomez@unizar.es (A.G.-C.); gervicen@unizar.es (G.V.-R.); 2 Faculty of Health and Sport Sciences (FCSD), Department of Physiatry and Nursing, University of Zaragoza, 22002 Huesca, Spain; 3 Centro de Investigación Biomédica en Red de Fisiopatología de la Obesidad y Nutrición (CIBEROBN), 28029 Madrid, Spain; marcela.gonzalez.gross@upm.es; 4 Red española de Investigación en Ejercicio Físico y Salud, EXERNET, University of Zaragoza, 50009 Zaragoza, Spain; eva.gesteiro@upm.es (E.G.); Ignacio.Ara@uclm.es (I.A.); 5 Faculty of Health Sciences (FCS), Department of Physiatry and Nursing, University of Zaragoza, 50009 Zaragoza, Spain; 6 HEME Research Group, University of Extremadura, 10003 Cáceres, Spain; jorgepg100@unex.es; 7 GENUD Toledo Research Group, Universidad de Castilla-La Mancha, 45071 Toledo, Spain; Sara.Vila@uclm.es; 8 CIBER of Frailty and Healthy Aging (CIBERFES), 28040 Madrid, Spain; ngusi@unex.es; 9 ImFine Research Group, Department of Health and Human Performance, Universidad Politécnica de Madrid, 28040 Madrid, Spain; 10 International Institute for Aging, 10003 Cáceres, Spain; 11 Physical Activity and Quality of Life Research Group (AFYCAV), Faculty of Sport Sciences, University of Extremadura, 10003 Cáceres, Spain; 12 Institute of Biomedicine (IBIOMED), University of León, 24071 León, Spain; jg.villa@unileon.es; 13 Unit of Sport Medicine, Cabildo of Gran Canaria, 35002 Gran Canaria, Spain; luisespinotoron@gmail.com; 14 Centro Universitario de la Defensa, 50090 Zaragoza, Spain

**Keywords:** aging, elderly, exercise, muscle, health

## Abstract

The aims of the present study were (1) to describe the changes in physical fitness during an 8 year follow-up in a large sample of Spanish adults aged 65 or over that are initially engaged in organized physical activity (OPA), (2) to compare fitness changes according to different age groups (65 to 69 vs. 70 to 74 vs. ≥75 years-old), (3) to evaluate the independent and combined effects of changes in OPA engagement and sitting time (ST) on physical fitness. A total of 642 (147 males) non-institutionalized over 65 years-old participants completed the EXERNET battery fitness tests and completed a validated questionnaire from which information regarding OPA and ST were collected. All participants completed evaluations in 2008–2009 and in 2016–2017. An impairment of fitness-related variables happens after 65 years of age in both males and females, with the older participants (≥75), showing the largest decreases. Males who continued performing OPA demonstrated lower decreases in balance, leg flexibility and agility when compared to those who stopped performing OPA during the follow-up. Females who continued performing OPA demonstrated lower decreases of all variables except for balance when compared to those who stopped performing OPA during the follow-up.

## 1. Introduction

Aging, which is defined as an age-dependent or age-progressive decline in intrinsic physiological function, entails a decrease in both cognitive [1] and physical functioning [2]. Regarding the latter, a decrease in fitness levels will determine the health and autonomy of older adults as different studies have shown that cardiorespiratory fitness [3] and muscle power [4] are determinants of physical functioning and consequently of independence in elderly subjects. Moreover, low physical fitness levels have been associated with higher health care costs [5], sudden cardiac death [6] or an increased number of falls [7] in this population. It will therefore be of critical importance to assess and establish determinants of healthy aging in older adults.

A large body of evidence has focused on the importance of physical activity and exercise as determinants of physical fitness and consequently healthy aging in older adults [2,8,9], with systematic reviews showing the positive effects of both resistance [9], aerobic [10] and multicomponent [11] interventions. Nonetheless, almost all interventions have a low ecological validity as most participants stop training when the intervention is ended, returning to initial values shortly after. On the other hand, observational studies do not necessarily modify the behavior of the participant, with a recent meta-analysis [12] demonstrating that higher levels of physical activity increase the odds of healthy aging by 39%. The mentioned meta-analysis [12] included 23 studies, although mostly from USA, Canada and Australia, with only 3 studies from Europe, all developed in England. Due to the differences between countries and the effect that environmental factors can play on physical activity and thus fitness, there is a clear need of performing longitudinal studies evaluating the effects of regular physical activity on fitness in the elderly in other European countries. Moreover, most studies evaluating the effects of physical activity on older adults use heterogeneous samples with wide age ranges and include both active and inactive participants. Nevertheless, some researchers advocate for only including participants that have been exercising for many years in studies aiming to evaluate human aging [13]. 

Finally, most studies only evaluate physical activity or exercise without considering sedentary behaviors (defined as sitting, reclining or lying down during prolonged periods) which are usually increased in this population, and have been linked to a decrease in physical fitness [14] and increased risk of frailty [15] obesity [16] and all-cause mortality, especially in Europe [17]. 

Therefore, the present study aims (1) to describe the changes in physical fitness during an 8 year follow-up in a large sample of Spanish adults aged 65 or over that are engaged in organized physical activity (OPA); (2) to compare the evolution of different age groups (65 to 69 vs. 70 to 74 vs. over 75 years-old) in order to test if there is a critical decline at a specific age group; (3) to evaluate the independent and combined effects of changes in OPA engagement and sitting time (ST) on physical fitness.

We hypothesize that independently of the age group, all participants will decrease their fitness levels (all variables), and that larger decreases will be found in the oldest groups. Both OPA and ST will be determinants of this evolution.

## 2. Methods

### 2.1. Study Sample

The study was carried out in the framework of the longitudinal elderly EXERNET study (Exernet Elder 3.0); a multi-center study performed between 2008 and 2009 (baseline) and 2016 and 2017 (follow-up). In order to be included in the study, participants had to be over 65 years and non-institutionalized in the first evaluation. In 2008, a representative sample of Spanish seniors was evaluated in 6 different regions across Spain. From an initial total sample of 3093 participants, 2987 performed the fitness assessment. From these, 2364 performed OPA and were selected for the present study. One center could not perform the follow-up due to lack of funding and therefore, the sample was reduced by 400 participants, passing from 2364 to 1964 eligible older adults. There were 236 detected deaths between 2008 and 2016, leaving a total of 1728 participants that were contacted in 2016. Due to several reasons (change of residence or city, did not answer the phone, became dependent and could not attend the follow-up or was not willing to undertake the evaluation), 1055 participants were not able to attend the follow-up. Consequently, 673 participants completed both evaluations. From these, 31 participants did not attend the day when fitness was assessed; therefore, 642 participants (147 males) fulfilled the inclusion criteria for the present manuscript, which represents 27% of the initial sample of 2364 participants who completed the fitness tests and were engaged in OPA.

Written informed consent was obtained from all the included participants. The protocol of the study was performed in accordance with the Helsinki Declaration of 1964 (revised in Edinburgh 2000 and Fortaleza, 2013) [18] and was approved by the Clinical Research Ethics Committee of Aragón (18/2008) for the baseline and by the Hospital Universitario Fundación de Alcorcón (16/50) for the follow-up.

### 2.2. Demographic Characteristics, OPA and ST

Data for all participants was registered through an interview by which researchers completed a validated questionnaire [19]. The questionnaire included several questions regarding OPA and ST, and for the present study the following questions from both evaluations were used:

1. How many hours do you usually spend sitting per day? The question covered any activity in which the person had to be sitting (i.e., watching television, reading, sewing, etc.) and it referred to the present time. The question was answered by 610 participants at both baseline and follow-up.
  Hours sitting per day were used to classify subjects into non-sedentary (<4 h/day) and sedentary (≥4 h/day). The cut-off points to define this sedentary behavior (SB) are based on receiver operating characteristic curves carried out with the same sample and reported in a previous study [20].
  According to the answer to the first question, participants were divided into two groups:
  - NON-SEDENTARY: A group of participants who had never been sedentary or had showed a positive change (passing from sedentary at baseline to non-sedentary in the follow-up).
  - SEDENTARY: A group of participants who were sedentary in both evaluations or showed a negative change (were non-sedentary at baseline but were in the follow-up).

The second question used to classify participants was:

2. Are you currently engaged in organized physical activity? The question covered any organized physical activity understood as a collective guided and supervised activity that was developed by an instructor. All participants that answered YES at baseline were included in the study (642 participants).
  According to the answers to the OPA question, participants were classified as:
  - ALWAYS OPA: Performed OPA at both baseline and follow-up.
  - STOPPED OPA: Performed OPA at baseline but not at follow-up.

Four groups (610 participants) were established using these questions based on the answers given at baseline and in the follow-up:

1. OPA-ACTIVE: Performed OPA longitudinally and were never sedentary or stopped being sedentary (223 females and 62 males).
2. OPA-SEDENTARY: Performed OPA longitudinally and were always sedentary or started being sedentary (160 females and 45 males).
3. SEDENTARY-INACTIVE: Stopped performing OPA and were always sedentary or started being sedentary (60 females and 16 males).
4. NON-SEDENTARY-INACTIVE: Were not sedentary longitudinally or stopped being sedentary and stopped engaging in OPA (34 females and 10 males).

### 2.3. Fitness Tests

Physical fitness was assessed with the EXERNET battery which includes tests from the Senior Fitness Test battery and the Eurofit Testing Battery [14]. The performed tests were:

- Anthropometry: body weight, height and body mass index (BMI)
- One leg balance test: to evaluate static balance.
- Thirty-second chair stand test: to evaluate lower extremities strength.
- Arm curl test: to evaluate upper extremities strength.
- Chair sit-and-reach test: to evaluate lower extremities flexibility.
- Back scratch test: to evaluate upper extremities flexibility.
- Eight-Foot up-and-go test: to evaluate agility.
- Brisk walking test: to evaluate walking speed.
- Six-Minute walk test (6MWT): to assess endurance capacity.

All the tests are explained in detail elsewhere [21]. The tests were all performed at baseline and follow-up by trained researchers.

### 2.4. Statistical Analyses

Data were checked for normality using the Shapiro–Wilks normality test and visual inspection of the histograms. Although balance, agility and walking speed scores were not normally distributed the central limit theorem was applied and all data were treated as normally distributed. This approach was selected as both *t*-tests and *F*-tests used in this research have shown to perform well when applied to both normal and non-normal distributions in large sample sizes [22].

In order to evaluate if there were important differences between the selected sample for the present study (642 participants who had complete data) and the measured sample in the first evaluation (2364 participants who had data for the first assessment and were engaged in OPA), we compared demographic and anthropometric characteristics (age, weight, height and BMI) of both samples with independent *t*-tests.

Regarding the final included sample in the present study (642 participants), dependent sample *t*-tests were performed to evaluate differences between the baseline and follow-up for anthropometric and demographic continuous variables, while chi-square tests were developed to evaluate changes in categorical data.

Participants were grouped according to age (at baseline) into three age groups (65–69.9 years: YOUNGER; 70–74.9 years: MID; and ≥75 years: OLDER). In order to evaluate differences in fitness variables between the two evaluations and to test differences between age groups, a repeated measures analysis of variance (ANOVA) was performed to check differences among the evolution of fitness variables of the three age groups. When significant groups were observed by time interactions, further contrasts were performed to determine between which groups the interactions emerged.

A repeated-measures analysis of covariance (ANCOVA) was performed to evaluate the differences between the OPA groups after adjusting by age. Another age-adjusted ANCOVA was developed to compare the ST groups. The same analysis was performed when dividing the group into four groups according to the combination of OPA and ST. This was only done for the females due to the low number of participants in each group in males (*n* = 10 for the NON-SEDENTARY-INACTIVE group and *n* = 16 for the SEDENTARY-INACTIVE group). The repeated measures ANCOVA were replicated adjusting by walking time, but due to the fact that results did not significantly change in most variables, only results for age adjustment are reported in tables and figures.

Effect size statistics using partial eta squared (ηp2) for repeated measures are reported. The effect sizes were considered small (0.01–0.06), medium (0.06–0.14) or large (>0.14) [23]. Statistical significance was set at *p* < 0.05.

For all analysis, the Statistical Package for the Social Sciences (SPSS) version 22.0 for Mac OS X (SPSS Inc., Chicago, IL, USA) was used.

## 3. Results

### 3.1. Retention Rates and Differences between Samples

The retention rate for the present longitudinal study was very low with only 27% of the initially measured participants presenting complete data in the follow-up.

When comparing demographic and anthropometric characteristics (age, weight, height and BMI) of the initial sample (2364 who completed the fitness tests and were engaged in OPA) with the final included sample (642 participants), we found that females included in the present study showed a lower weight and BMI (Supplementary Table S1) and both males and females included in the present study were younger (Supplementary Table S1).

When stratifying results according to age groups, the age differences were only maintained for the OLDER group (Supplementary Table S1).

### 3.2. Descriptive Characteristics

The main characteristics of the sample are presented in Table 1, separately for males and females. Both males (baseline 70.3 ± 4.3 years) and females (baseline 70.7 ± 4.4 years) significantly decreased height (*p* < 0.05) during the 8-year follow-up maintaining the same BMI. Females also decreased body weight (*p* < 0.05).

### 3.3. Physical Fitness

#### 3.3.1. Physical Fitness Changes during the 8-Year Period Stratifying by Sex

All fitness variables were significantly impaired during the 8-year period (all *p* < 0.05; Table 2). The highest effect sizes were found for the changes in endurance capacity that in the 8-year period decreased from 612 to 528 m in males (large effect; partial eta squared (ηp^2^) 0.516) and from 537 to 462 m in females (large effect; ηp^2^ 0.474). Changes in walking speed (from 14.4 to 16.5 s, ηp^2^ 0.307 in males and from 17.0 to 20.0 s in females, ηp^2^ 0.326) and balance (from 42.3 to 27.5 s, ηp^2^ 0.400 in males and from 30.5 to 19.0 s, ηp^2^ 0.269 in females) also showed large effect sizes.

#### 3.3.2. Physical Fitness Changes during the 8-Year Period Stratifying by Sex According to Age Group

##### Males

Group-by-time interactions were found for balance, agility, walking speed and endurance capacity (Table 3; all *p* < 0.05). Further contrasts showed that for balance the OLDER group showed larger decreases than the YOUNGER group (21.8 vs. 11.6 s decrease, respectively; group-by-time interaction (*GxT*) *p* < 0.05). Regarding agility, the YOUNGER group showed a lower deterioration (0.5 s) when compared to the MID (1.1 s; *GxT p* < 0.001) and OLDER groups (1.5 s; *GxT p* < 0.05). For endurance capacity, both the YOUNGER (walked 69 m less; *GxT p* < 0.001) and MID (walked 82 m less; *GxT p* < 0.05) showed lower decreases in walked meters when compared to the OLDER group (walked 129 m less). Finally, for walking speed, significant group-by-time interactions emerged among the three groups with the YOUNGER group presenting a lower deterioration in walking speed (increase of 1.2 s) when compared to the MID (increase of 2.4 s; *GxT p* < 0.05) and the OLDER groups (increase of 4.1 s; *GxT p* < 0.001). Differences also emerged between the MID and the OLDER group (*GxT p* < 0.05).

##### Females

Group-by-time interactions were found for leg strength, arm strength, agility, walking speed and endurance capacity (Table 3; all *p* < 0.05). Further contrasts showed that the OLDER group presented larger decreases for both leg strength and arm strength when compared to the YOUNGER and MID groups. The OLDER group presented the largest deterioration for agility (1.5 s slower), walking speed (4.9 s slower) and endurance capacity (walked 94 m less), when compared to both the MID (agility 1.1 s slower; *GxT p* < 0.001/walking speed 2.8 s slower; *GxT p* < 0.001/endurance capacity 75 m less; *GxT p* = 0.059) and YOUNGER (agility 0.7 s slower; *GxT p* < 0.001/walking speed 2.2 s slower; *GxT p* < 0.001/endurance capacity 65 m less; *GxT p* < 0.05) groups. No differences were found between the YOUNGER and MID groups.

A significant impairment of physical fitness was found for all age groups (Table 3).

#### 3.3.3. Physical Fitness Changes Stratifying by Sex According to Change in Organized Physical Activity

##### Males

**Figure 1:**
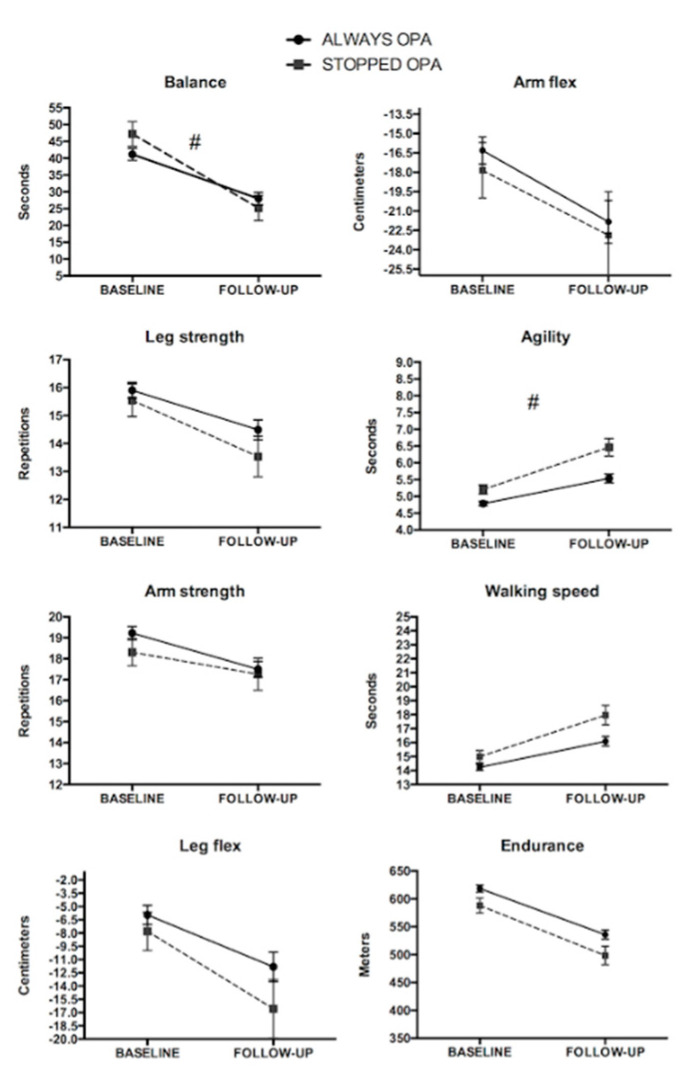
Fitness changes according to organized physical activity (OPA) engagement during the follow-up in males. ^#^ Significant group-by-time interaction. Both groups showed a significant decrease from baseline to follow-up (*p* < 0.05), except for the arm strength of the group that stopped OPA (*p* = 0.100).

Participants who continued performing OPA demonstrated lower decreases in balance and agility when compared to those who stopped performing OPA during the follow-up (all *GxT p* < 0.05; Figure 1).

This was the only analysis that changed after adjusting by walking time as the group-by-time interaction found for agility became non-significant (*p* = 0.076).

##### Females

**Figure 2:**
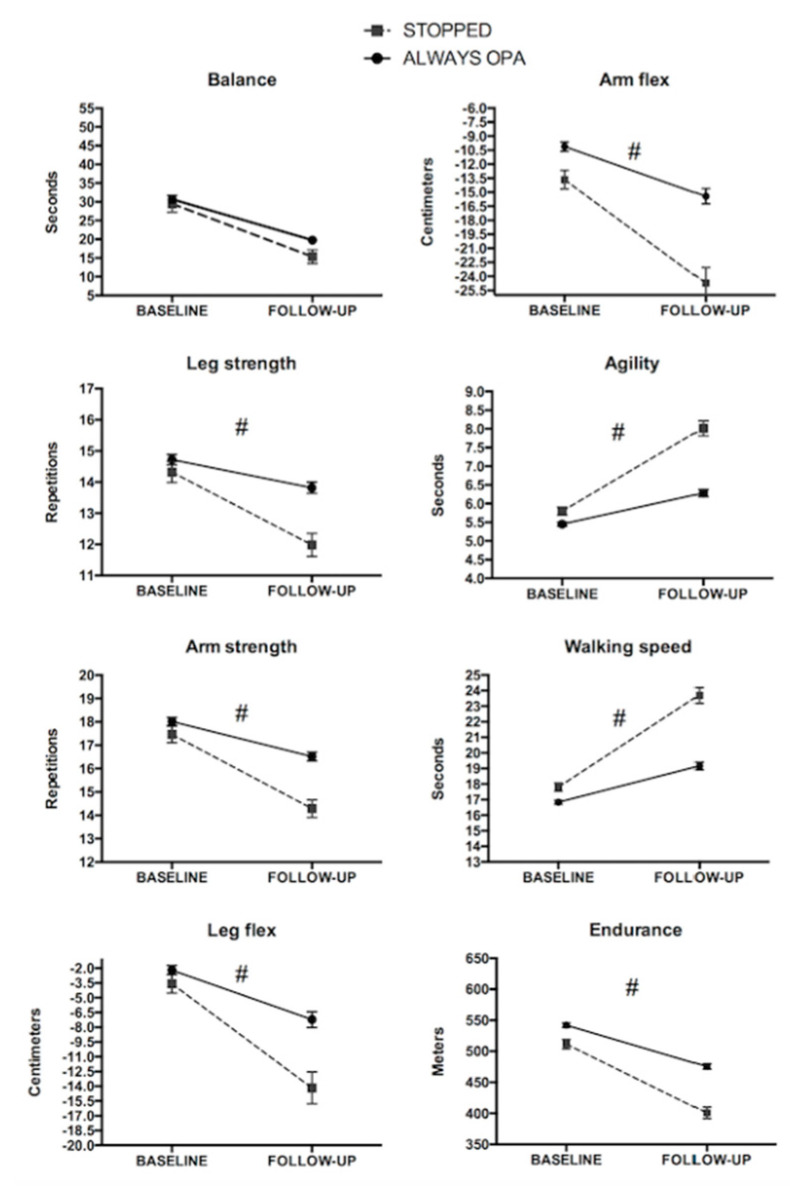
Fitness changes according to OPA engagement during the follow-up in females. ^#^ Significant group-by-time interaction. Both groups showed a significant decrease from baseline to follow-up (*p* < 0.05).

Participants who continued performing OPA demonstrated lower decreases of all variables except for balance when compared to those who stopped performing OPA during the follow-up (all *p* < 0.05; Figure 2).

#### 3.3.4. Physical Fitness Changes Stratifying by Sex According to Sitting Time

##### Males

**Figure 3:**
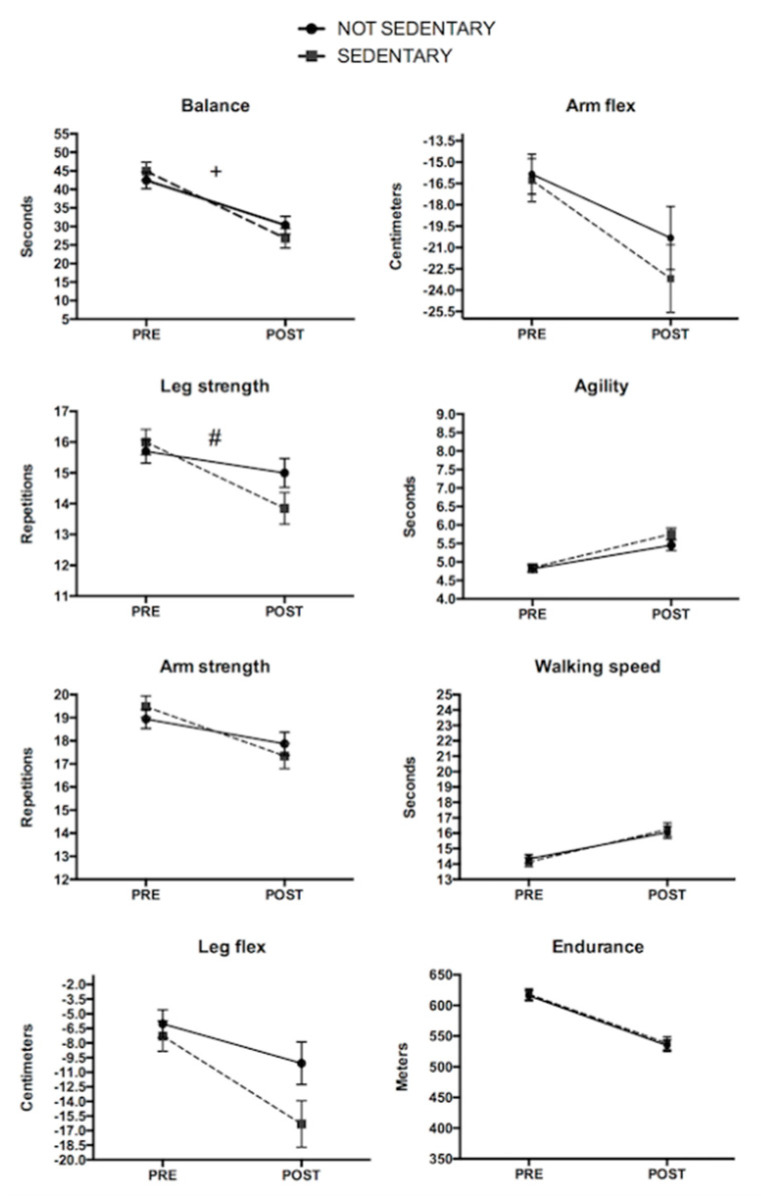
Fitness changes according to sitting time (ST) during the follow-up in males. ^#^ Significant group-by-time interaction. ^+^ Trend towards significant group-by-time interaction (*p* = 0.061).

SEDENTARY participants demonstrated higher decreases in balance (*GxT p* = 0.061; Figure 3), and leg strength (*GxT p* < 0.05; Figure 3) when compared to the NON-SEDENTARY group during the follow-up. Both groups significantly impaired all physical fitness variables from baseline to follow-up (all *p* < 0.05; Figure 3) except for leg strength that did not decrease significantly in the NON-SEDENTARY group.

Both groups showed a significant decrease from baseline to follow-up (*p* < 0.05), except for the leg strength of the NOT-SEDENTARY group.

##### Females

**Figure 4:**
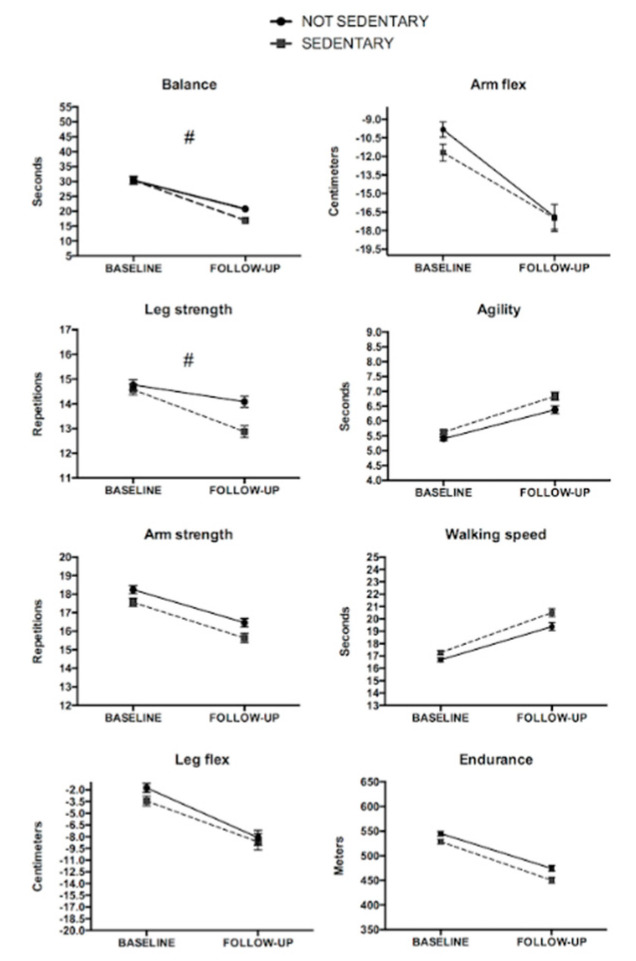
Fitness changes according to ST during the follow-up in females. ^#^ Significant group-by-time interaction. Both groups showed a significant decrease from baseline follow-up (*p* < 0.05).

SEDENTARY participants demonstrated higher decreases in balance and leg strength when compared to the NON-SEDENTARY group during the follow-up (all *GxT p* < 0.05; Figure 4). Both groups significantly impaired all physical fitness variables from baseline to the follow-up (all *p* < 0.05; Figure 4).

#### 3.3.5. Physical Fitness Changes Stratifying by Sex According to Change in Organized Physical Activity and Sedentary Behaviors.

##### Females

**Figure 5:**
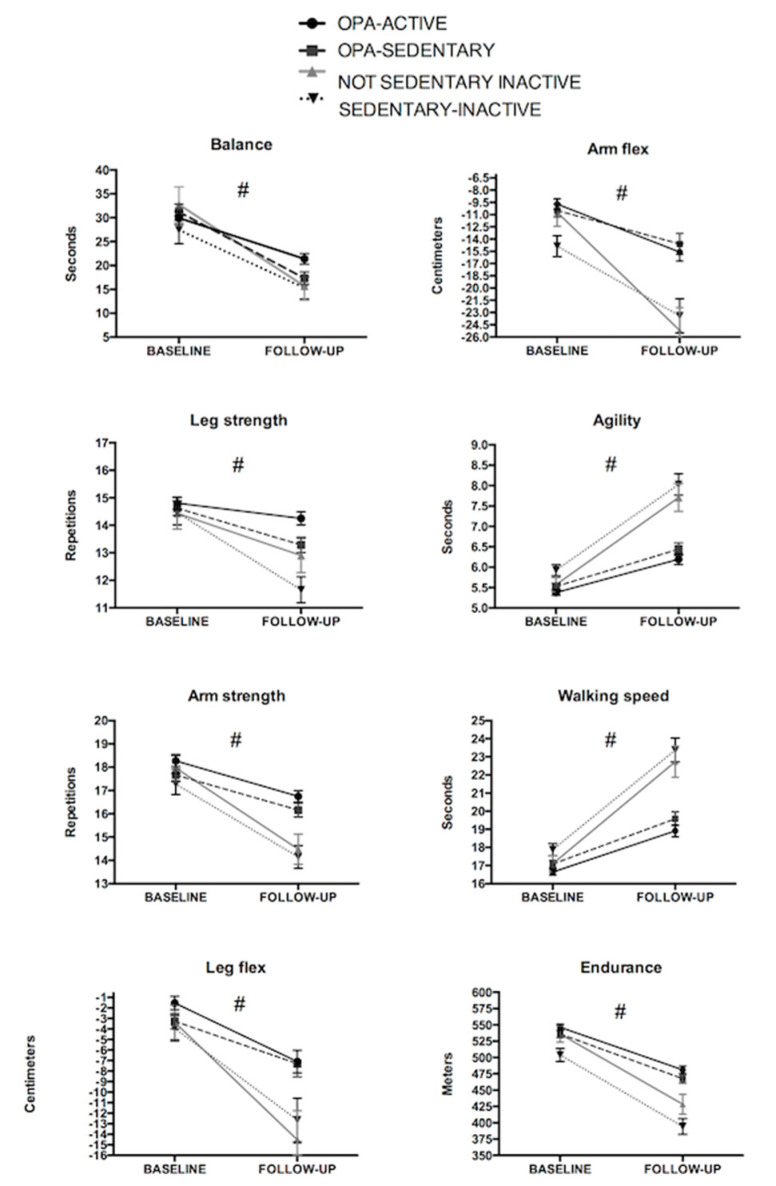
Fitness changes according to OPA engagement and sedentary behaviors. ^#^ Significant group-by-time interaction among the 4 groups. All groups showed a significant decrease from baseline to follow-up (*p* < 0.05).

Significant group-by-time interactions were found for all the fitness variables (all *p* < 0.05; Figure 5).

##### OPA-ACTIVE vs. OPA-SEDENTARY

When performing further pairwise comparisons, the OPA-ACTIVE group presented lower decreases in balance and leg strength when compared to the OPA-SEDENTARY group (both *GxT p* < 0.05; Figure 5).

##### OPA-ACTIVE vs. NON-SEDENTARY INACTIVE

The NOT-SEDENTARY INACTIVE group presented a larger decrease in balance, arm strength and flexibility, agility, speed and endurance capacity when compared to the OPA-ACTIVE group (all *GxT p* < 0.05; Figure 5).

##### OPA-ACTIVE vs. SEDENTARY-INACTIVE

The SEDENTARY-INACTIVE group presented a larger decrease in leg and arm strength, agility, walking speed and endurance capacity when compared to the OPA-ACTIVE group (all *GxT p* < 0.05; Figure 5).

##### OPA-SEDENTARY vs. NON-SEDENTARY INACTIVE

The NON-SEDENTARY INACTIVE group presented a larger decrease in arm strength, leg and arm flexibility, agility, walking speed and endurance capacity when compared to the OPA-SEDENTARY group (all *GxT p* < 0.05; Figure 5).

##### OPA-SEDENTARY vs. SEDENTARY-INACTIVE

The SEDENTARY-INACTIVE presented a larger decrease in leg and arm strength and flexibility, agility, walking speed and endurance capacity when compared to the OPA SEDENTARY group (all *GxT p* < 0.05; Figure 5).

##### NON-SEDENTARY INACTIVE vs. SEDENTARY-INACTIVE

The SEDENTARY-INACTIVE presented a larger decrease in leg strength when compared to the NON-SEDENTARY INACTIVE group (*GxT p* = 0.06; Figure 5).

## 4. Discussion

To the best of our knowledge, this is one of the first studies evaluating the effects of staying engaged with or stopping organized physical activity, considering sitting time during an 8-year follow-up in males and females aged 65 or over. The main findings are: (1) an impairment of fitness levels happens after 65 years of age in both males and females, (2) the older participants (75 or older), showed the largest decreases, and (3) both OPA and ST are critical to the evolution of physical fitness and can modulate the negative effects of aging on health related fitness.

The decline in all the fitness variables was expected, as ageing is a natural and inevitable process with several previous studies reporting a decrease of fitness with age [24,25]. Similarly to previous studies [26], we found that this decline was not linear, as both males and females in the oldest group (≥75 years) showed the largest decreases for agility, walking speed and endurance capacity. This decrease is determinant, as the maintenance of the previously mentioned fitness variables has been associated with physical independence [27], which will be critical in this age group as it will determine institutionalization, frailty and/or death [15,28].

Surprisingly, different evolutions for the strength variables seemed to emerge between sexes with the three male age groups showing a similar leg and arm strength decline while for females, the older group showed a larger decline when compared to the younger groups. Previous studies also assessed the evolution of muscle strength during aging and compared sexes with literature showing inconsistent results as some studies found similar declines for males and females [26], while others described different declines [26,29,30]. Although we do not have a clear explanation for the sex differences in the evolution of muscle strength found in our study, they could partly be explained by changes in muscle fiber number and area, as Essen-Gustavsson [31] reported a larger decline for older women in type I (men 15% and women 25%) and type II fiber area (men 19% and women 45%) that could affect muscle strength. Moreover, the European health survey of 2014 [32], reports that both males and females show a decrease in the compliance to the physical activity guidelines passing from 32.9% males and 25.6% females complying in the 65 to 74 year age group, to 20.3% males and 12.4% females complying in the 75 to 84 year age group and finally to a 11.4% males and 4.6% females complying in the over 85 age group. It is obvious that although both males and females decrease the levels of physical activity, these levels are reduced to a higher extent in females in Spain, which could also help to explain the higher decreases in the fitness variables found in the female older age group.

Opposite results were found when analyzing balance, as females showed a similar decrease in all age groups, while for males the decline was not linear with the OLDER males (≥75 years) showing larger decreases when compared to the YOUNGER males (65–69.9 years). Our results are in line with those of Puszczaloska-Lizis et al. [33], who compared different balance-related variables in males and females of three different age groups (60–69 years, 70–79 years and 80–90 years). Although the researchers did not evaluate the differences between age groups within sexes, they did find that most of the statistically significant sex-related differences were observed for the 70–79 and 80 to 90 year groups (favoring the female group), which would correspond with the OLDER group of the present study, suggesting that the decline in postural stability is steeper in men than in women. Balance in the elderly can be affected by several factors, such as change of posture with consequent shift of the center of gravity, impaired vision, vestibular signal problems or loss of muscle strength. In our sample, the OLDER males showed similar muscle strength losses when compared to the other age groups and therefore the balance problems could emerge from different pathways, such as somatosensory systems of the foot and ankle joint, as well as joint stiffness.

When evaluating the effect of OPA on fitness, the results were clear and showed that those who performed OPA during the 8-year follow-up period presented lower decreases in most physical fitness variables, when compared to those who stopped performing OPA. Although the effects were clear for both sexes, larger differences were found for females, suggesting that women might obtain greater benefits from OPA than men, as for the latter no differences were found between activity groups for arm and leg strength, arm flexibility, walking speed and endurance capacity. The lack of differences for arm and leg strength and walking speed (which is influenced by leg strength) are in line with previous studies performed in our laboratory [34] and by other researchers such as Martin et al. [35] who found that higher levels of activity were associated with greater muscle strength in women but not in men or Bassey et al. [36], who found that muscle strength was associated with leisure activity in women but not in men. For the present sample, both males and females at the beginning of the study were all engaged in OPA, which mainly consisted of “keep fit gym classes”, which were guided by an instructor who developed the same class for males and females. Consequently, it is possible that the classes were not strenuous enough to stimulate strength improvements or maintenance in the male group. Therefore, it is possible that those males who stopped, showed similar decreases in muscular strength than those who continued the program. It is also possible that males who stopped participating in OPA continued performing physical activity individually or with friends but without an instructor or monitor (not organized), which could also improve or maintain their fitness levels and well-being [37].

ST also seemed to be a critical variable that influenced physical fitness although to a lesser extent than OPA, as only balance and leg strength were affected, with the SEDENTARY group showing larger significant decreases in both variables in males and females than the NON-SEDENTARY group. These findings are of critical importance and underline the relevance of previous cross-sectional studies [14] that suggested the need for incorporating new programs and activities that change lifestyle and reduce total ST per day or break up prolonged periods of ST in this population. When combining OPA and ST, again the results were clear, showing that the OPA-ACTIVE group characterized by less than 4 h ST and engagement in OPA during the 8-year period was the group that showed the smallest decreases of physical fitness during follow-up. ST showed to be a critical factor for physical fitness, as when comparing those with the same level of OPA but different ST patterns (OPA-ACTIVE vs. OPA-SEDENTARY and SEDENTARY-INACTIVE vs. NON-SEDENTARY INACTIVE), the results favored those who spent less than 4 h sitting, suggesting that ST and not only the lack of physical activity, is critical to fitness in the elderly. Although the cut-off point of 4 h was established in a previous study [20] and might seem like very little daily sitting time, Harvey et al. [38] described that estimated sedentary behaviors from self-reporting are approximately half of that measured objectively. Therefore, our cut-off point would probably be around eight real sitting hours during the day.

The present findings are important not only because they describe the effect of different aging behaviors on fitness, but also because of the direct and indirect effects that changes on fitness have on quality of life of older adults, as physical fitness has an important role in the functionality, mobility, autonomy, health and welfare of this population. Moreover, decreases in fitness can induce other problems. For example, decreases in muscle strength have been shown to be negatively associated with bone loss in both men and women [26] which in turn will increase the risk of fracture. Additionally, cardiorespiratory fitness decreases have been associated with white matter lesions which are major determinants of cognitive decline and disability in old age [39]. Consequently, maintaining high physical fitness levels will reduce the risk of suffering both physical and mental problems.

Although the study included a large sample, the follow-up retention percentage (27%) was very low. Bhamra et al. [40] developed a systematic review evaluating attrition rates of older people (>55 years) in longitudinal studies suggesting that age increases the problem of attrition, as older participants have increased death rates, and an increased probability of becoming frail and dropping out of studies. These factors could partly explain why the included sample in the present study was younger than the whole initial sample that we measured in the first evaluation and could also partially explain the low retention rates as a vast amount of participants in the study changed residence or became dependent/frail and were not able to attend the follow-up tests. Nonetheless, it is important to notice that two critical factors that have been associated with attrition rates in older adults (retirement and social participation) were controlled in the present study as all participants were retired when the study started (retirement has been negatively associated with attrition [40]) and all participants were initially involved in OPA (not belonging to a club or association is a predictor of attrition in older participants [40]). Further studies should take this into account when performing power calculations, especially when fitness is going to be assessed, as similar studies that just perform telephone or household interviews might not have the same problems [37].

The present study presents several strengths such as a large sample size of participants who were all initially engaged in OPA or the assessment of physical fitness through different specific fitness tests. Nevertheless, there are five main limitations that should be acknowledged. Firstly, the aforementioned retention rates were very low and could have biased the sample although we do not think they have affected the external validity of the study. The second limitation is that we did not differentiate among OPA, with some participants engaged in “keep fit” guided classes and others in Pilates or yoga activities. Thirdly, data to classify participants according to their OPA engagement, walking and sitting time was collected through questionnaires, and consequently participants were classified according to their perceptions of the OPA they performed and their walking and ST. Moreover, these questionnaires were only completed twice by each participant (at baseline and follow-up) and therefore participants with different evolutions (e.g., a man that stopped OPA a month after the baseline and a man that stopped OPA seven years after the baseline), would be classified into the same group (following the previous example, both would be classified into the STOPPED-OPA group). The fourth factor to take into account is that although we controlled for walking time, which is one of the most popular activities in older adults, we did not register other non-organized physical activities that could have influenced our results (e.g., gardening, swimming, running, etc.). Finally, we did not control for other factors that could also affect fitness such as change of marital status and diet. Regarding the latter, recent studies have suggested that nutrition can enhance the impact of exercise on muscle mass and consequently on strength in older adults [41], and we would therefore suggest that future studies evaluating fitness decline in older adults take diet into account.

## 5. Conclusions

To conclude, OPA and ST are critical to physical fitness in the elderly, both independently and combined. Consequently, people over 65 willing to maintain or reduce as little as possible their physical fitness should stay engaged in OPA and avoid ST as much as possible. Different results were found for males and females which suggest that exercise interventions focusing on improving physical fitness in older adults should try to maintain agility, walking speed and endurance capacity and should be sex-specific. Exercise programs designed for males should include exercises aiming to maintain and improve balance while exercise programs designed for females should focus on muscle strength maintenance. ST should be reduced trying to avoid more than 4 h of self-reported ST.

## Figures and Tables

**Table 1:** Descriptive characteristics of the participants divided by sex for both the baseline and follow-up evaluations.

Variables		Males			Females		
		Baseline	Follow-Up	*p* Value	Baseline	Follow-Up	*p* Value
Anthropometric characteristics							
Age (years)		70.3 ± 4.3	77.8 ± 4.6	<0.001	70.7 ± 4.4	78.3 ± 4.7	<0.001
Weight (kg)		78.3 ± 9.5	77.7 ± 9.8	0.920	67.5 ± 10.0	66.5 ± 10.7	<0.001
Height (cm)		165.6 ± 3.1	164.6 ± 5.9	<0.001	152.5 ± 5.8	151.5 ± 5.9	<0.001
BMI (kg/m^2^)		28.6 ± 3.1	28.6 ± 3.2	0.967	29.0 ± 4.0	29.0 ± 4.3	0.811
Physical activity and sedentarism							
OPA	yes (%)	147 (100%)	118 (80.3%)		495 (100%)	397 (80.2%)	
	no (%)	0 (0%)	29 (19.7%)		0 (0%)	98 (19.8%)	
Daily sitting time	<1 h	1 (0.8%)	1 (0.8%)	0.426	10 (2.1%)	2 (0.4%)	<0.001
	1–2 h	12 (9.0%)	9 (6.8%)		54 (11.3%)	47 (9.9%)	
	2–3 h	42 (31.6%)	31 (23.3%)		147 (30.8%)	100 (21.0%)	
	3–4 h	40 (30.1%)	31 (23.3%)		125 (26.2%)	108 (22.6%)	
	4–5 h	19 (14.3%)	33 (24.8%)		76 (15.9%)	109 (22.9)	
	>5 h	19 (14.3%)	28 (21.1%)		65 (13.6%)	111 (23.3%)	
Daily walking time	<1 h	35 (24.5%)	27 (18.9%)	0.032	166 (33.9%)	198 (40.5%)	<0.001
	1–2 h	75 (52.4%)	74 (51.7%)		244 (49.9%)	222 (45.4%)	
	2–3 h	27 (18.9%)	29 (20.3%)		68 (13.9%)	40 (8.2%)	
	3–4 h	3 (2.1%)	9 (6.3%)		7 (1.4%)	14 (2.9%)	
	4–5 h	3 (2.1%)	3 (2.1%)		2 (0.4%)	7 (1.4%)	
	>5 h	-	1 (0.7%)		2 (0.4%)	8 (1.6%)	
Sedentary	Yes	38 (28.6%)	61 (45.9%)	0.011	141 (29.6%)	220 (46.1%)	<0.001
	No	95 (71.4%)	72 (54.1%)		336 (70.4%)	257(53.9%)	

Data for 495 females and 147 males, except for daily sitting (completed by 477 females and 133 males) and daily walking time (completed by 489 females and 143 males). BMI = Body mass index; OPA = Organized physical activity; Sedentary = Spends more than 4 h sitting.

**Table 2:** Evolution of the physical fitness variables.

Variables	Males			Females		
	Baseline	Follow-up	ηp^2^	Baseline	Follow-up	ηp^2^
Balance (Seconds)	42.3 ± 20.4	27.5 ± 22.2	0.400 *	30.5 ± 20.9	19.0 ± 17.6	0.269 *
Leg strength (repetitions)	15.8 ± 2.3	14.3 ± 3.9	0.144 *	14.7 ± 3.2	13.5 ± 3.6	0.099 *
Arm strength (repetitions)	19.0 ± 3.8	17.5 ± 4.2	0.139 *	17.9 ± 3.4	16.1 ± 3.9	0.182 *
Leg flexibility (centimetres)	−6.3 ± 11.7	−12.8 ± 17.8	0.147 *	−2.5 ± 9.4	−8.6 ± 16.0	0.137 *
Arm flexibility (centimetres)	−16.6 ± 11.4	−22.0 ± 17.8	0.107 *	−10.8 ± 9.9	−17.3 ± 16.6	0.161 *
Agility (seconds)	4.9 ± 0.8	5.7 ± 1.6	0.307 *	5.5 ± 1.0	6.6 ± 2.2	0.264 *
Walking speed (seconds)	14.4 ± 2.3	16.5 ± 4.1	0.307 *	17.0 ± 2.6	20.0 ± 5.3	0.326 *
Endurance (meters)	612.6 ± 74.1	528.6 ± 98.9	0.516 *	536.6 ± 72.1	462.1 ± 93.1	0.469 *

ηp^2^ = partial eta squared, * = Significant differences from baseline to follow-up

**Table 3:** Physical performance stratified by age groups.

Title		Males		Within	G*x*T	Females		Within	G*x*T
		Baseline	Follow-up	ηp^2^	*p*	Baseline	Follow-up	ηp^2^	*p*
Balance (seconds)	65–70y	49.5 ± 17.8	37.8 ± 2.8	0.184 *	0.045	35.2 ± 21.1	25.4 ± 20.0	0.110 *	0.211
	70–75y	34.4 ± 21.0	17.8 ± 18.9	0.206 *		28.7 ± 20.2	15.4 ± 13.2	0.144 *	
	>75y	34.2 ± 19.6	12.3 ± 15.0	0.205*		22.7 ± 18.8	10.5 ± 12.4	0.077 *	
Leg strength (repetitions)	65–70y	16.1 ± 2.9	15.2 ± 3.6	0.032 *	0.117	14.8 ± 3.0	14.0 ± 3.4	0.024 *	0.008
	70–75y	15.8 ± 2.9	13.5 ± 4.0	0.108 *		14.5 ± 3.3	13.4 ± 3.6	0.030 *	
	>75y	15.2 ± 3.3	13.3 ± 4.1	0.048 *		14.6 ± 3.4	12.4 ± 3.8	0.072 *	
Arm strength (repetitions)	65–70y	19.6 ± 3.5	18.2 ± 3.6	0.062 *	0.833	18.5 ± 3.5	17.0 ± 3.7	0.059 *	<0.001
	70–75y	18.9 ± 3.0	17.1 ± 5.1	0.058 *		17.6 ± 3.5	16.0 ± 3.9	0.054 *	
	>75y	17.5 ± 4.0	15.7 ± 3.4	0.035 *		17.3 ± 2.9	14.3 ± 3.5	0.121 *	
Leg flexibility (centimetres)	65–70y	−5.8 ± 12.3	−12.9 ± 20.0	0.102 *	0.822	−1.8 ± 8.9	−8.6 ± 17.6	0.080 *	0.600
	70–75y	−5.5 ± 10.7	−10.7 ± 12.9	0.028 *		−2.0 ± 9.9	−7.2 ± 14.0	0.037 *	
	>75y	−8.9 ± 11.2	−15.4 ± 17.2	0.032 *		−4.6 ± 8.9	−10.8 ± 15.5	0.035 *	
Arm flexibility (centimetres)	65–70y	−15.6 ± 10.7	−22.2 ± 20.9	0.088 *	0.224	−9.6 ± 8.9	−16.1 ± 17.8	0.081 *	0.902
	70–75y	−16.8 ± 11.9	−18.7 ± 13.4	0.004		−10.8 ± 9.7	−16.9 ± 15.3	0.055 *	
	>75y	−19.2 ± 12.7	−26.8 ± 12.5	0.041 *		−13.4 ± 11.9	−20.3 ± 15.7	0.044 *	
Agility (seconds)	65–70y	4.7 ± 0.7	5.2 ± 0.8	0.078 *	<0.001	5.3 ± 0.9	6.0 ± 1.4	0.062 *	<0.001
	70–75y	4.9 ± 0.7	6.0 ± 1.8	0.194 *		5.6 ± 1.1	6.6 ± 2.0	0.102 *	
	>75y	5.3 ± 0.9	6.7 ± 2.1	0.215 *		5.8 ± 1.0	7.9 ± 3.0	0.239 *	
Walking speed (seconds)	65–70y	13.9 ± 2.1	15.1 ± 2.5	0.082 *	<0.001	16.4 ± 2.2	18.7 ± 4.0	0.114 *	<0.001
	70–75y	14.7 ± 2.4	17.1 ± 4.7	0.168 *		17.2 ± 2.5	20.0 ± 1.1	0.134 *	
	>75y	15.4 ± 2.5	19.4 ± 4.8	0.258 *		18.0 ± 3.2	22.9 ± 7.9	0.224 *	
Endurance (seconds)	65–70y	635.1 ± 64.2	566.1 ± 70.1	0.288 *	0.005	549.0 ± 72.2	483.6 ± 86.1	0.237 *	0.014
	70–75y	594.0 ± 74.7	511.9 ± 98.1	0.241 *		536.5 ± 68.5	461.8 ± 82.6	0.238 *	
	>75y	579.2 ± 80.9	450.6 ± 117.6	0.337 *		509.8 ± 71.4	415.6 ± 107.4	0.228 *	

* = Significant within group changes (*p* < 0.05). Within = Changes within group; G*x*T = Group-by-time interaction; ηp^2^ = partial eta squared.

## Supplementary Materials

The following are available online at https://www.mdpi.com/1660-4601/17/12/4312/s1, Table S1: descriptive and anthropometric characteristics of the initial (whole sample) and the final included sample (study sample).
