# Supplementary material for: The Effects of Age, Organized Physical Activity and Sedentarism on Fitness in Older Adults: An 8-Year Longitudinal Study
Source: Int J Environ Res Public Health. 2020 Jun 16;17(12):4312. doi: 10.3390/ijerph17124312 (PMC7345727; doi:10.3390/ijerph17124312)
Supplement: Supplementary file 1 [file ijerph-17-04312-s001.pdf]

		Males		Females	
		Study sample	Whole sample	Study sample	Whole sample
All ages included	Age (y)	70,26±4,29	72,12±5,45*	70,71±4,43	71,97±5,18*
	Weight (kg)	78,33±9,48	77,18±10,68	67,54±10,01	68,72±10,71*
	Height (cm)	165,40±5,89	164,93±6,65	152,53±5,84	152,59±5,81
	BMI (Kg/m2)	28,67±3,18	28,34±3,41	29,05±4,06	29,55±4,36*
65-70y	Age (y)	66,92±1,33	66,87±1,49	66,60±1,50	66,66±1,51
	Weight (kg)	78,38±10,29	78,69±10,93	68,59±10,59	70,30±11,07
	Height (cm)	166,04±5,41	166,48±6,28	153,65±5,97	154,13±5,75
	BMI (Kg/m2)	28,38±3,29	28,31±3,50	29,07±4,29	29,63±4,50
70-75y	Age (y)	71,95±1,32	71,98±1,29	71,99±1,29	72,07±1,33
	Weight (kg)	79,76±9,32	78,15±11,40	67,67±9,85	69,02±10,69
	Height (cm)	164,59±6,93	164,41±6,52	152,40±5,68	152,68±5,58
	BMI (Kg/m2)	29,66±3,47	28,95±3,55	29,18±4,20	29,66±4,47
<75y	Age (y)	77,26±2,51	78,78±3,47*	77,22±2,08	78,23±3,03*
	Weight (kg)	76,12±6,76	74,25±8,99	65,11±8,59	66,59±9,97
	Height (cm)	164,83±5,42	163,50±6,86	150,38±5,24	150,65±5,54
	BMI (Kg/m2)	28,08±1,94	27,76±3,08	28,79±3,31	29,35±4,09

Supplementary table 1. Descriptive and anthropometric characteristics of the initial (whole sample) and the final included sample (study sample). *p<.05
